# Effects of In-Situ Filler Loading vs. Conventional Filler and the Use of Retention-Related Additives on Properties of Paper

**DOI:** 10.3390/ma13225066

**Published:** 2020-11-10

**Authors:** Maria Emiliana Fortună, Andrei Lobiuc, Lucian-Mihai Cosovanu, Maria Harja

**Affiliations:** 1 Inorganic Polymers Department, “Petru Poni” Institute of Macromolecular Chemistry, Aleea Gr. Ghica Voda 41A, 700487 Iasi, Romania; fortuna.maria@icmpp.ro; 2 Human Health and Development Department, “Stefan cel Mare” University, Universitatii Str. 13, 720229 Suceava, Romania; 3 Integrated Center for Research, Development and Innovation in Advanced Materials, Nanotechnologies, and Distributed Systems for Fabrication and Control, “Stefan cel Mare” University, Universitatii Str. 13, 720229 Suceava, Romania; lucian.cosovanu@usm.ro; 4 Faculty of Chemical Engineering and Environmental Protection, Gheorghe Asachi Technical University of Iasi, 73, Prof.dr.doc. D. Mangeron Blvd., 700050 Iasi, Romania; maria_harja06@yahoo.com

**Keywords:** additive systems, conventional loading, in-situ fiber loading, mechanical properties, optical properties

## Abstract

In the present paper, aspects concerning the obtained and characterization of additive systems used for maximizing filler retention, and the effects on paper properties, were investigated. The effects of retention additives over properties of paper, containing fibers from in-situ loading (IS-CCP), were analyzed against the effects of additives over properties of paper containing fibers from conventional loading, obtained by the addition of calcium carbonate in precipitated form (CCP). The physico-mechanical properties were analyzed by various analyses and investigations: calcium carbonate content, X-ray diffraction, scanning electron microscopy (SEM) images, optical and mechanical properties, in order to develop the best systems of retention additives for obtaining higher retention loads for making paper with high content of nano-filler material. The obtained results reveal that at the same level of calcium carbonate content, all paper samples with in-situ loading had higher the optical and mechanical properties than the paper obtained by conventional loading in all cases the additives studied. For all studied properties, nanoparticles had a positively influence over paper properties.

## 1. Introduction

The current demographic development, coupled with situations such as the current epidemic, raises a number of challenges in manufacturing industrial scale goods. As in other major industries, the paper industry is influenced by the actual pandemy [1]. The annual world paper consumption has reached at 423.3 million metric tons [2]. The changes in people’s lives will influence industrial production, having a major impact over pulp and paper industry. On one hand, although paper, writing and printing materials may decrease in demand, on the other hand an increasing demand for other paper products (personal hygiene, food packaging products, corrugated packaging materials, paper-based medicinal materials/devices/parts—medical specialty papers, masks etc.) is observed [3,4,5]; in this context, the diversification of papers types and improvement of their properties is of high interest [6,7,8].

The stages of paper manufacturing are: Material preparation (pulp manufacturing and bleaching), paper manufacturing, and fibers recycling [9]. Issues of cost and paper quality have determined papermakers in using mineral additives [10,11]. The use of functional additives, such as calcium carbonate as a filler and/or coating pigment has become common in neutral/alkaline thin paper manufacturing technology, with the purpose to improve properties, such as increasing opacity, brightness, water penetration control, and respectively, increase the wet and dry strength of the paper. At the same time, ever higher addition of filling material requires establishing additive systems for retention and dehydration corresponding to the neutral/alkaline environment of papermaking and of hydrodynamic conditions on the making machine [12]. Consequently, fillers play an important role in producing quality printing paper, and also in the economic efficiency of the papermaking system.

Nanoparticles could really induce an improvement in paper properties, especially in mechanical, electrical, heat resistance, radiation resistance, as a result of the proper dispersion of the nano-fillers in the matrix. The nanoparticles, (CaCO_3_, clay, SiO_2_, TiO_2_, ZnO), etc., have been used to make paper [13]. Of these, CaCO_3_ is attractive because of its low cost and high quality, but researches had as objectives increasing the interaction between the fiber and filler by surface-treated CaCO_3_ [14]. The different nanomaterials (NM) can be incorporated into the paper structure and allows paper products to be created with novel properties. The use of NMs in industrial papermaking nowadays mainly focuses on the use of inorganic pigments, minerals, ceramics and starch [15]. Final properties depend on shape, size and size distribution of fillers and coating pigments. The problems that can should be resolved for using nano-fillers at the industrial scale, are their poor retention and higher cost [10]. These disadvantages are resolved for the CaCO_3_ nano-fillers because of the availability of raw materials, the simplicity and synthesis low cost procedure. The nano-sized calcium carbonate has shown significant attention due to its remarkable properties and market demand in many industrial approaches [16,17]. The different methods for synthesis of CaCO_3_, with different properties, has been described, these depending on additive types and synthesis conditions [18,19].

The fillers can be entrapped mechanically into the network by filtration, or chemically by developing the aggregation mechanisms with the aid of chemical additives. Since the retention additives improve filler retention by aggregation of the particles, the efficiency of filler content concerning optical properties decreases. At the same time, the filler content has a strong negative impact on strength properties of paper [20,21].

In the literature, there are two common synthetic sizing agents who are used in neutral-alkaline paper making: of alkyl–dimercetene (AKD) and alkenyl succinic anhydride (ASA) [22,23]. Increasing the filler content and the fine material from pulp for thin paper has a negative influence on the efficiency of alkyl–dimercetene due to the high superficial surface entered in the system. It was shown that adding cationic starch improves sizing by preferential adsorption of alkyl–dimercetene particles in fibers, which leads to increased retention of the first pass of alkyl–dimercetene [24,25]. At the same time, the increase in the secondary fiber content needs diversification and an increase in additive consumption for controlling the processes and the quality of the finite product. One of the most usual retention systems with a single component uses a cationic polyacrylamide with relatively high molecular mass and charge density small-medium [11,26,27]. Cationic polymers are often added in the paper pulp due to their positive effects on retention, dehydration or paper resistance [10]. The molecular mass of the polymer is also very important because this depends mainly on the configuration of the polymer adsorbed on a surface [28]. For a low molecular mass, it is possible not to have the loops and ends of the polymer extended in solution, which is mainly responsible for the effective inversion of particle surfaces. Therefore, the polymeric chain will be less flexible and will adsorb itself in a relatively flat configuration, leading to the neutralization of the charge and not to its inversion. Wagberg and Lindstrom [29] have shown that, if polymer charge is high, interactions between polymer-surface result in a flatter adsorbed polymer configuration on the fiber surface, thus lowering its ability to formation polymer bridges. Additive systems using a component with inorganic nanoparticles like bentonite have the goal of preventing the formation of big flocs, which appear when a traditional mono-component or dual-component system is used. This allows equilibrium to be reached between the retention and dehydration processes without affecting paper formation. Currently, there are different types of nanoparticles, which in addition to silica colloidal and bentonite, include anionic or cationic synthetic soils [30,31]. Those developments opened new opportunities for effective application of nano-particles retention systems in obtaining fine paper with lower mass, but also of other sorts like over calendered papers [26].

Nano-fillers can be fixed onto fibers by various methods: Co-mixing with flocculation or fixation chemicals (conventional loading), or by direct “in-situ” precipitation via various precursors. In-situ precipitations determine a high filler retention [24] and more uniform distribution of filler material within the sheet. In order to improve optical properties with very low loss of strengths, optimum parameters must be established [19,32].

Conventional filler loading of paper consists in the dosing and uniform distribution of filler particles into fibre suspension, which are further incorporated in paper structure by retention during wet-web formation. In conventional loading paper, all factors that contribute to the improvement of optical properties produced a decrease in paper strength indexes. The limits of conventional loading become even more visible with low filler retention into paper sheet since, a large part of the filler particles are passing through the forming wire into the process water.

Some researchers in this area [33,34] demonstrated improvements in both optical and strength properties of paper as benefits of the in-situ loading method, besides a very high retention yield. Others [35,36] reported contradictory results on the increase in the mechanical resistance of paper through in-situ loading.

Literature data [33,34,36] points out the various benefits of in-situ loading method, such as improvements in the optical and strength properties of the paper, decrease in the environmental impact of papermaking by lowering the process water loading as a result of high filler retention, and by reducing of the energy consumption for the pulp stock preparation and waste water treatment.

At present, researchers look for new paper loading methods, which could lead to higher filler retention and a better relationship between the optical and strength characteristics.

In this sense, the identification of proper systems of retention nano-fillers for obtaining higher retention loads for papermaking with a high content of filling material, as well as the influence of different of additive systems over papers properties obtained using various fiber compositions: Cellulose of hardwood/cellulose of softwood loading by precipitation in-situ of calcium carbonate were analyzed comparatively, with the cellulose of hardwood/cellulose of softwood for which calcium carbonate was conventionally dosed, in order to gradually or at once conventional filling. It is expected that the high porosity and high surface area might contribute to strengthening the mechanical properties of paper; the approaches to maintain paper strengths at higher nano-filler levels are also considered. An improvement in the optical and strength properties of paper in using the in-situ method, besides a very high retention yield in all systems studied, was demonstrated.

## 2. Materials and Methods

*Materials:* hardwood fibers (H) and softwood fibers (S) were refined in a Valley Hollander at 30° SR (Schopper-Riegler degree); calcium chloride (for analysis, 96%); sodium carbonate (for analysis, 99.8%); alkyl–dimercetene emulsion (AKD) under the trade name—Aquapel 210D supplied by Hercules Co (AKD, constant addition to pulp of 10 kg/t) used as a sizing agent for paper; cationic starch—derived from corn, prepared by boiling at 90 °C, (CS, constant addition to pulp of 5 kg/t); cationic polyacrylamide supplied by Ciba Specialty Chemicals-Ciba^®^ Percol^®^ Co (Basel, Switzerland) (PAA, constant addition to pulp of 0.025%); bentonite (B, constant addition to pulp of 0.25%); anionics microparticles with commercial names Ciba^®^ Hydrocol ^®^ OT—an anionic modified bentonite used as component in systems or retention/dehydration.

*Methods:*

In-situ precipitation of calcium carbonate (IS-CCP) was performed by the double-exchange reaction between calcium chloride and sodium carbonate:

CaCl_2_ + Na_2_CO_3_ → 2NaCl + CaCO_3._ (1)

The bleached softwood fibers (S), refining to 30° SR, was dehydrated at 30% consistency and treated with solution of calcium chloride under stirring (100 rpm). After the addition was completed, the sodium carbonate solution is introduced continuing the stirring for another hour. The reaction proceeds at room temperature. The pulp washed in several steps for complete removal of the free calcium carbonate particles. The paper hand sheets were obtained on a Rapid-Köthen apparatus, at a standard basis weight of 70 g/m^2^.

*Calcium carbonate used for the conventional filling (CCP)* was obtained from solution of calcium chloride and sodium carbonate by mixing them in a reaction vessel under stirring, at 100 rpm for an hour. Calcium carbonate thus formed is filtered and washed with distilled water, then dried at 120 °C for 24 h.

*Conventional loaded fiber* stock was prepared by the dosing and uniform distribution of precipitated calcium carbonate into refined pulp suspension under stirring; the dosage of calcium carbonate was established at 30% after several retention tests to obtaining paper hand sheets with the same content of calcium carbonate as those of in-situ loaded fiber pulp [26,31].

*Experimental program*

**Figure 1:**
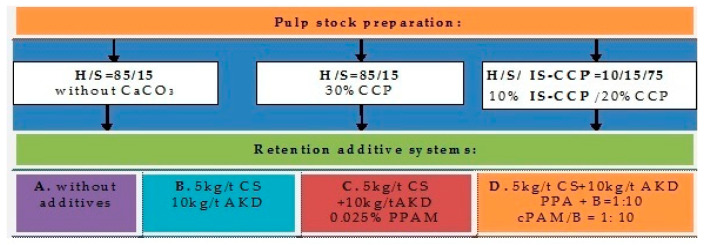
Experimental design/treatment methods.

The experimental program, realized at room temperature, involved the use of three types of pulp fibers (Figure 1):

- hardwood fibers/softwood fibers (H/S)—reference;
- hardwood fibers/softwood fibers with the constant addition of 30% precipitated calcium carbonate (H/S/30% CCP)—conventional loading;
- hardwood fibers/softwood fibers/softwood fibers with in-situ precipitation of calcium carbonate (10% IS-CCP)/20% CCP added for finally to obtain the same calcium carbonate content in pulp (30%), (H/IS-CCP/20%CCP)—in-situ loading.

Also, the effect of different additives on paper properties for the three types of pulp fibers obtained was investigated (Table 1). Therefore, there were obtained four series of experiments:

A. without additives;
B. cationic starch (CS) and alkyl–dimercetene emulsion (AKD);
C. cationic starch (CS), alkyl–dimercetene emulsion (AKD) and cationic polyacrylamide (PAA);
D. cationic starch (CS), alkyl–dimercetene emulsion (AKD), cationic polyacrylamide (PAA) and bentonite (B).

*Analyses:*

Paper sheets were conditioned under standard conditions (24 hrs at 23 °C and 50% relative humidity). Paper samples, before and after different treatments were analyzed using:

- Calcium carbonate content, according to Tappi Standard T413 [37];
- Water absorptiveness (CobbValue)—is a function of various characteristics of paper or board such as sizing, porosity, etc. according to toTappi 441;
- Air permeability (cm^3^g/cm^2^·min·m^2^), measured, according to SR ISO 5636-1:2001 at a 1 kPa pressure;
- The breaking length of paper sheets (Lr [km]), was measured with the Instron instrumentation (according to Tappi Standard T413);
- Burst factor (kPa m^2^/g) measure with the Schopper-Dale instrumentation (Tappi 494);
- Opacity and brightness measured with spectrophotometer L&W Elrepho 2000, according to ISO 2471;
- X-ray diffraction remains the main method for the identification of atom arrangement in minerals. X-ray analysis was applied to determine the polymorphic form of calcium carbonate particles retained in paper sheets obtained from in-situ loaded fibre pulps. X-ray diffraction patterns of calcium carbonate particulates were obtained on a D8 ADVANCE, Bruker-AXS apparatus, equipped with a transmission type goniometer, using nickel-filtered, CuKα radiation (λ = 1.5418 Å) at 36 kV; the goniometer was scanned stepwise every 0.10° from 10° to 60° in the 2θ range; X-ray diffraction was performed only for paper sheets obtained from washed pulps;
- Scanning electron microscope (SEM) (VEGA/TESCAN instrument with an accelerating voltage of 30 kV) to identify the localization of calcium carbonate crystals into the cellulose fibre structure (lumen and fibre wall pores);
- Particles size distribution of precipitated calcium carbonate with Laser diffraction particle size analyzer (Sald-7001/Shimadzu Scientific Instrument).

All experiments were performed with 5 batches of product for the different variants of paper. For each parameter, means and standard errors were calculated, while statistical significance was assessed using analysis of variance—ANOVA, followed by a post-hoc Tukey test, for *p* = 0.05. As such, the pool of values for each parameter was 5 samples (n = 5).

## 3. Results and Discussion

### 3.1. Scanning Electron Microscopy

**Figure 2:**
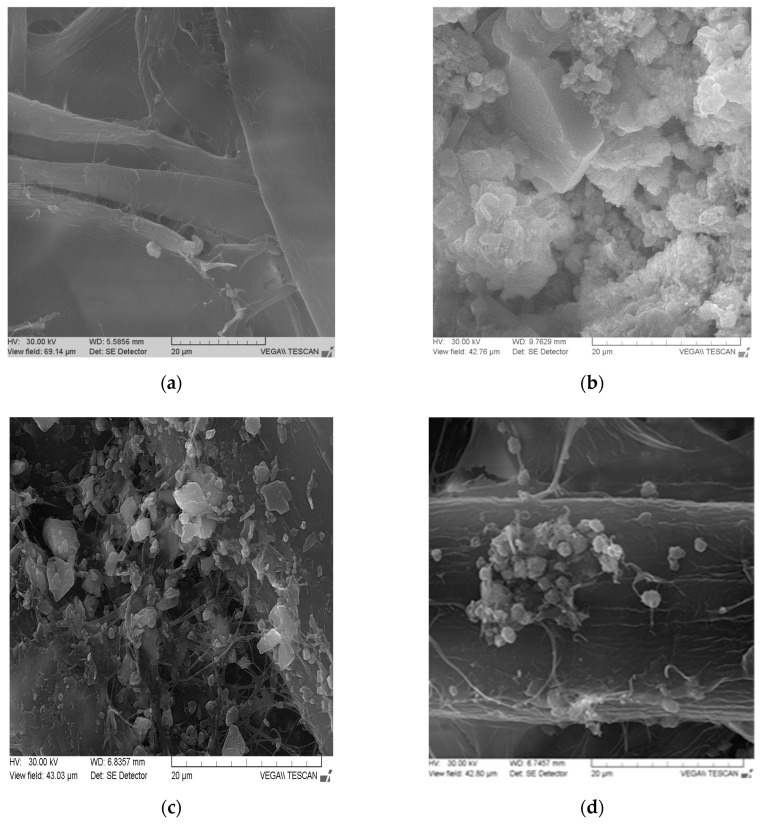
SEM images of paper samples, obtained by conventional and respectively, in-situ loading with precipitated calcium carbonate. (**a**) Without loading. (**b**) CaCO_3_ obtained from CaCl_2_/Na_2_CO_3_. (**c**) In-situ loading. (**d**) Conventional loading.

SEM images of the paper surface were taken to investigate the effect of particle size, shape and surface modification on the retention and distribution CaCO_3_ within the paper sheets [31,38]. The neat paper exhibits a porous structure and consists of cellulose fibers with smooth surface (Figure 2). The results from Figure 2 indicated that in-situ CaCO_3_ crystallization leads to the formation of nanocrystals, and the samples have higher retention, nanoparticles formed having uniform distribution than the conventional loading. In cases of conventional loading of CaCO_3_ for obtaining cellulose papers, the porous structure is quite similar to neat cellulose paper, but agglomerations of CaCO_3_ on the surface of fibers can be observed.

Particle size depends of different parameters, fact demonstrated in previously papers [18,39,40,41]. In Figure 2, it can observed that calcium carbonate obtained in-situ have particles sizes up to 0.01 µm, with no aggregations.

**Figure 3:**
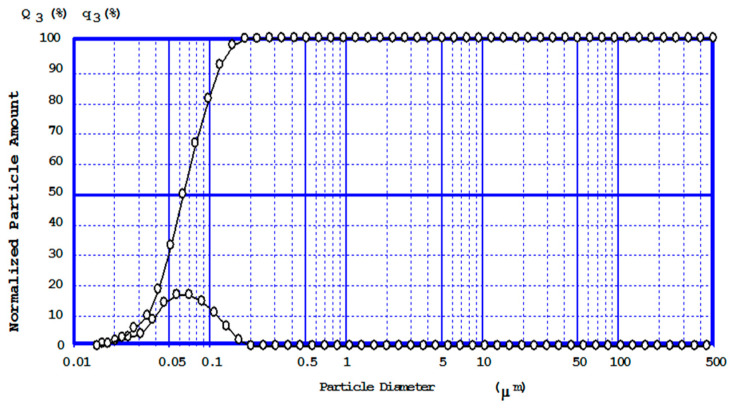
Particles size distribution of precipitated calcium carbonate (particle diameters and cumulative curve).

The particle size distribution of the particles was determined using a laser technique. The samples were dispersed by ultrasound for 10 min, and distilled water was used as the dispersion medium. During particle sizing, it was found that the particles were highly agglomerated and ultrasound can break up the particles effectively, as seen in Figure 3. Sizing showed that CaCO_3_ synthesized by double exchange reaction has the d_50_ of 0.05 µm, and particles with narrow diameters.

### 3.2. X-ray Analysis

**Figure 4:**
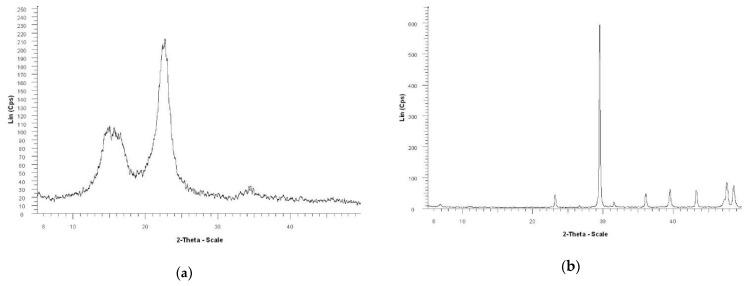

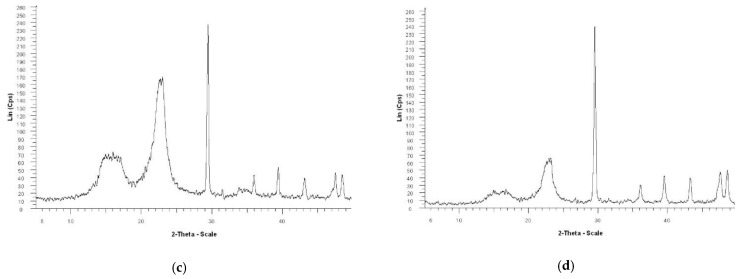
X-ray diffraction of paper samples and raw materials: (**a**) without loading; (**b**) CaCO_3_ obtained from CaCl_2_/Na_2_CO_3_; (**c**) in-situ loading; (**d**) conventional loading.

In this study, X-ray analysis was applied to determine the polymorphic form of calcium carbonate particles retained in paper sheets obtained from in-situ loaded fibre pulps. As reported in the literature, [37,38] calcite (Figure 4b) is rhombohedral in shape, R3c, with axes a = 4.99 Å as well as c = 17.06 Å. In the origin and also in layers, Ca atoms every c/6 along with c were recorded. Planar CO_3_ groups are perpendicular to c.

By X-ray diffraction the calcite and vaterite CaCO_3_ crystals precipitated in the wall and/or lumen of the fibers was observed (Figure 4c). Regardless of the obtaining method, a significant reflection of calcite at 2θ = 29.3°, the (104) plane, and at 2θ = 39.4°, the (113) plane can be observed, in accordance with literature [14,42].

### 3.3. Calcium Carbonate Retention

**Figure 5:**
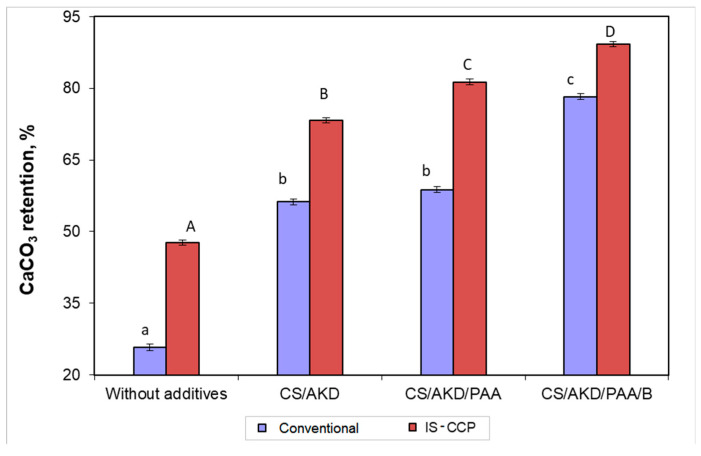
CaCO_3_ retention for different additive systems in relation to paper composition. (different letters within the same method—small letters for conventional and capitals for IS-CCP designate statistical difference, *p* < 0.05).

The retention efficiency was determined by calculating the ratio of calcium content in the paper to the quantity of calcium carbonate added initially in the pulp. Figure 5 shows the evolution of calcium carbonate retention using different additive systems. The Figure 5 shown that, in the all analyzed systems the calcium carbonate retentions is higher for the in-situ loading method comparatively with conventional loading. In the case of paper obtained without additives, retention efficiency of calcium carbonate is very small (up to 50% for in-situ method) and increases by adding retention additives in the pulp. Using cationic starch in papermaking is based on its property of flocculation of fibers and fine material particles and filling materials, due to its charge weakly cationic, thus improving their retention in the paper [43].

Cationic starch is a multifunctional additive in the paper machine wet, being extensively used in systems alkaline environment, for retention of sizing agent (alkyl–dimercetene), as well as the agent for increasing resistance of paper [28].

A first attempt to increase the retention yield of calcium carbonate was by treating the calcium carbonate suspension with PAA. We noticed that, adding PAA affects calcium carbonate retention, for in-situ loading method, the increase is major, reaching 81.33% compared with 58.8% in the case of the conventional loading.

The influence of the anionic nano-particles based on bentonite was established. In this case the PAA was added first into the system. Bentonite particles, with sizes between 300 nm × 100 nm × 5 nm, act as a tying agent between floccons’ surface [44]. Bentonite had positive effects on retention (Figure 5) having the capacity of adsorbing anionic materials and colloidal anionics on its surface, contributing to the cleaning system. On the other hand, the bentonite reduced softwood and sticky materials in the system with a further decrease in paper breaks, as well as improve the functioning and productivity of the paper machine [45]. The flocculation process of PAA/B system is mainly based on electrostatic interactions. The addition of PAA, at fibre surface a positive charge is generated, which will interact further with the negative charge on the bentonite surface. The floccons can be formed by the interaction of polymer chains with fibres or by the interaction between polymer chains, bentonite and fibres.

The results also show that, when the cationic polyacrylamide (PAA) system is combined with bentonite (B), the efficiency increases, resulting in a significant increase of the retention yield of calcium carbonate in the paper (Figure 5). Nanoparticles of calcium carbonate obtained in-situ and nano-bentonite determined the retention values over 90%, that mean significant cost reduction but and environmental positive effect (small quantities of CaCO_3_ are lost by washing). Another significant advantage of nano-CaCO_3_ could be reduced equipment wear, as compared to conventional ground carbonate where the size of the filler leads to increased wire use of the machines used.

### 3.4. Air Permeability

**Figure 6:**
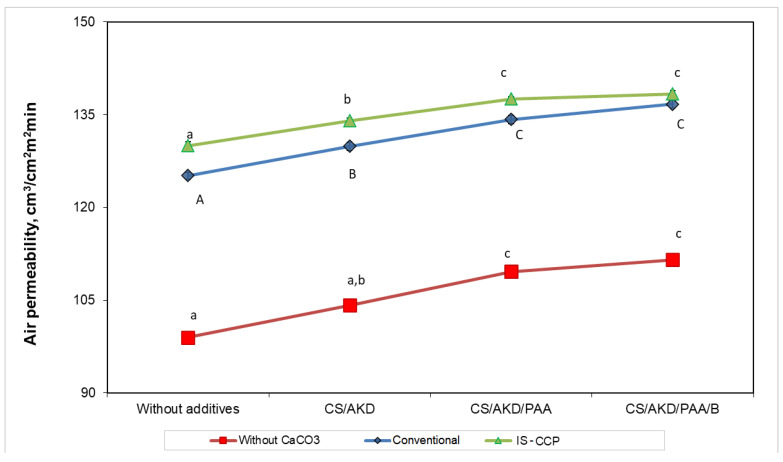
Air permeability for different additive systems in relation to paper composition (different letters within the same method—small letters for conventional and capitals for IS-CCP designate statistical difference, *p* < 0.05).

The porous structure of paper has an important role in paper efficiency regarding multiple of its further applications. The porosity of paper is usually correlated with the air resistance and it reverse, air permeability [38]. Since the level of aggregation of pulp influences the porous structure of the paper, air permeability measurements can give an indication of possible mechanisms of aggregation developed by various additive systems and their effect on paper quality. Air permeability (Figure 6) increases by adding additives in the pulp, highest increase is shown for the paper sheets obtained by the in-situ loading method and PAA+B, which shows that nanoparticles formed contribute to regulating porosity of composite materials. Furthermore, it is considered that filler particles allow larger spaces between fibers, decreasing their compactness and thereby leading to increased bulkiness of the material.

Air permeability tests showed that the application of the filling formulations with different additives increased air permeability significantly. The highest air permeability was achieved for the paper filled in-situ with the addition of PAA and B. The lowest and highest averages of air permeability were observed for the control sample.

### 3.5. Effects of Retention Additives on Paper Properties

#### 3.5.1. Cobb Value

The Cobb test is essential as it tests the ability of paper to resist the penetration of water and quantity of water absorbed by the surface of cardboard evaluates the quality of paper over a given time.

**Figure 7:**
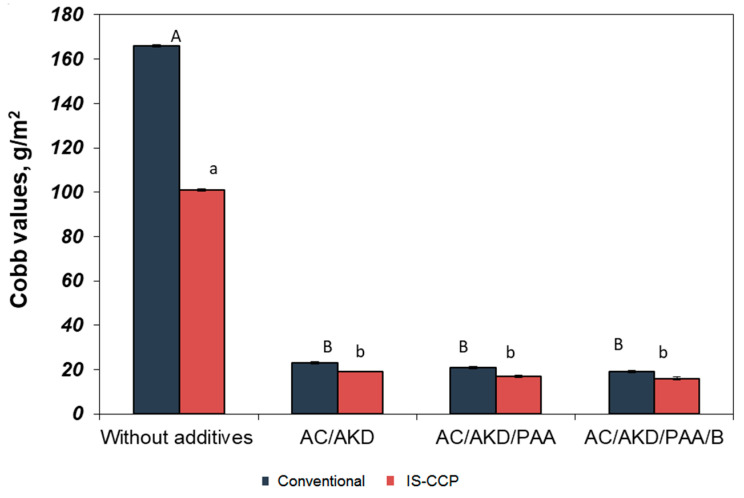
Cobb values for different additive systems in relation to paper composition (different letters within the same method—small letters for conventional and capitals for IS-CCP designate statistical difference, *p* < 0.05).

The Cobb_60_ values for different additive systems in relation to paper composition are depicted in Figure 7.

By addition of cationic starch and alkyl–dimercetene emulsion (AKD) in the pulp, Cobb_60_ values registers a significant decrease, therefore the sizing degree of paper sheets is improving. It is thought that cationic starch, used for retention of the emulsion sizing, helps to attach alkyl–dimercetene (AKD) particles on cellulose fibers with a negative charge, increasing total AKD quantity retained on paper [46]. Calcium carbonate frequently used in printing/writing papermaking can affect sizing by reducing AKD retention, but also by increasing the surface or by reducing the average diameter of the paper pores. The systems with nanoparticles determine the significant positively effect over paper quality.

#### 3.5.2. Optical Properties

**Figure 8:**
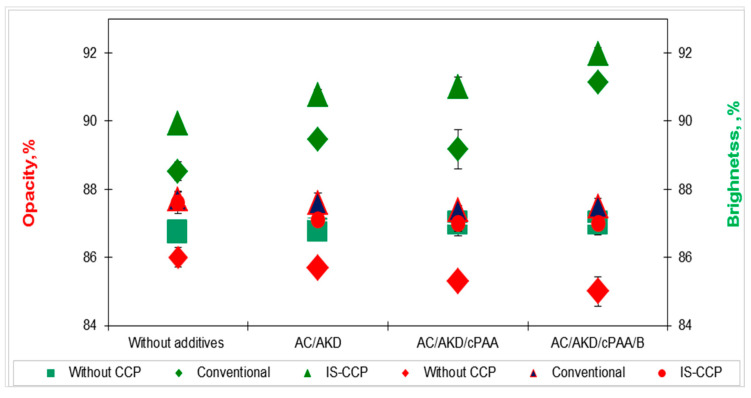
Evolution of brightness (♦, ▲, ●) and opacity (■, ♦, ▲) for different additive systems in relation with paper composition.

The evolution of brightness and opacity of paper sheets for studied systems are shown in Figure 8. The brightness does not increase significantly, although the quantity of calcium carbonate retained in paper, as opposed opacity is positively influenced by additives addition.

No retention system among those studied produces a significant decrease in the degree of whiteness, and the efficiency of the filling method, respectively (Figure 8), because the low load density and small additions of additives do not cause changes in the ionic balance of the system. However, an interesting effect was obtained when using bentonite. Cationic polyacrylamide, as the only component, shows a slight decrease in the degree of whiteness, which can mainly be due to particle aggregation (formation of large flocons) and possible interactions of the filler with cationic groups of polyacrylamide. In combination with bentonite, the increase in cPAM addition leads to a slight improvement in the degree of whiteness, due to the formation of smaller floccons and probably due to the fact that bentonite prevents the direct interaction between the filler and the cationic group. The highest increase arises in paper sheets obtained by in-situ loading, the effect caused by an increase in retention of calcium carbonate within the paper, which leads to an increase in optical unevenness of paper structure. The presence of nano-sized filler induces a higher surface area of particles and higher filler to fiber ratio, therefore paper brightness increases. Another mechanism, which explains improved optical properties of papers prepared with CaCO_3_, is the elevated light scattering capacity of rhombohedral calcite particles, which, in turn, is owed to uniformly sized particles and bulkiness.

Figure 8 shows that the opacity decreases with increasing brightness, but the tensile strength is increased. This development indicates that the reduction in opacity is mainly due to the physical relationship between brightness and opacity and not to structural changes in the paper, which can occur by varying the content and distribution of calcium carbonate in the paper.

#### 3.5.3. Mechanical Strength Properties

**Figure 9:**
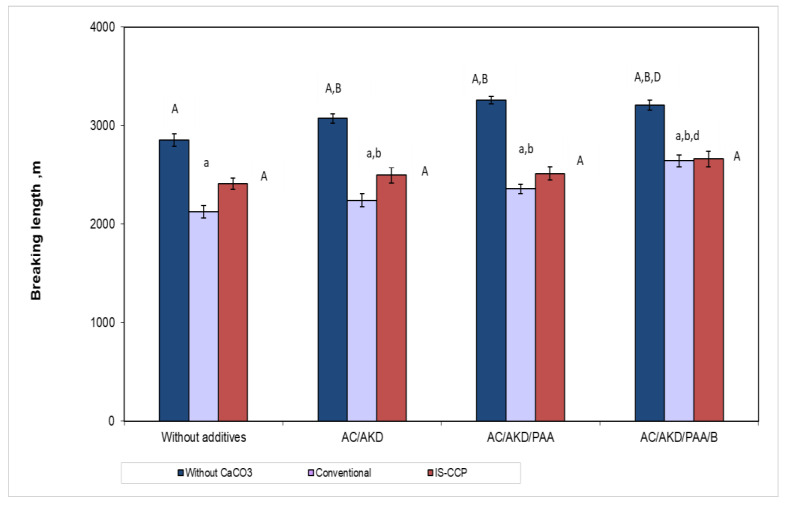
Breaking length for different additive systems in relation with paper composition (different letters within the same method—capitals for w/o CaCO_3_ and, respectively IS-CCP and small letters for conventional designate statistical difference, *p* < 0.05).

**Figure 10:**
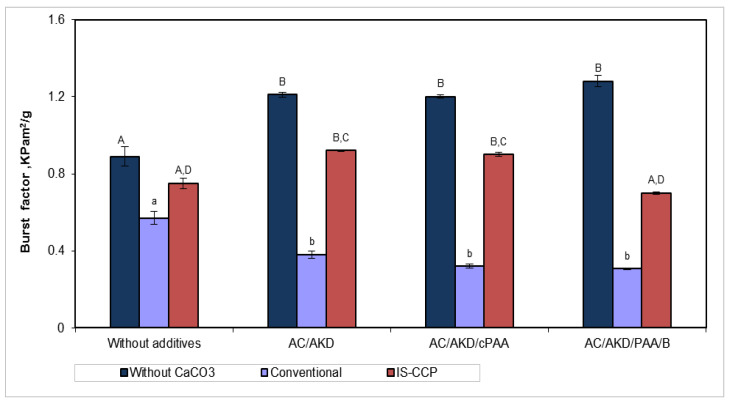
Burst factor for various additive systems in relation with paper composition (different letters within the same method—capitals for w/o CaCO_3_ and, respectively IS-CCP and small letters for conventional designate statistical difference, *p* < 0.05).

The mechanical properties (Figure 9 and Figure 10) are significantly influenced by additives addition. The increase of the strength properties for paper sheets obtained by in-situ loading (in presence of nanoparticles) can be observed. In Figure 9 can see that burst resistance decreases with additives addition, respectively calcium carbonate retention, thus presence of nano-filler having a relative positive effect. In conventional loading case, CaCO_3_ particles are higher and are retained in inter fiber spaces, reducing the contact capacity may explain this influence. In the absence of any retention additive, the opacity and tensile strength of the paper are adversely affected.

The presence of nano-filler did not have a significant effect on the breaking length, but the opposite was observed for burst factor, as seen in Figure 10. It can be shown that, as retention additives are added, both breaking length and burst factor increase, those being higher for the paper with the content of fibers loading by in-situ precipitating of calcium carbonate.

## 4. Conclusions

The effects of cellulose fibers loading by precipitation in-situ of calcium carbonate comparatively with conventional paper loading respectively, by adding precipitated calcium carbonate into fibre stock, over the properties of printing paper using different retention additive systems, were researched.

The data shows that calcium carbonate retention in paper is higher for fibre stock with in-situ loading compared with conventional loading. The evaluation of the effects on paper properties indicate that the utilization of fibres with in-situ loading controls the printing paper properties, especially with regard on the relationship between optical and strength properties, in all cases of the additive system studied.

The mechanical strength properties of paper obtained by in-situ loading method are improved, over those of the paper obtained by conventional loading, where calcium carbonate particles are larger and agglomerated, interposed between fibres. This change is likely to reduce printing capability. The analysis of the X-ray diffraction patterns and SEM images show that, regardless of the in-situ precipitation method applied, the calcium carbonate particulates present a typical calcite diffraction pattern, precipitation occurring in both the lumen and the wall pores of the cellulose fibres. However, these analyses evidenced significant differences among the in-situ precipitation methods as to particle sizes and their distribution into the fibre wall and lumen.

Cellulose fibres loaded by in-situ precipitation of calcium carbonate determine the formation of structures with higher porosity, compared with conventional loading method, and retention additives contribute to adjusting the porosity.

## Figures and Tables

**Table 1:** Sample preparation.

Pulp Stock Preparation			
Samples	Without CaCO_3_ (H/S)	Conventional (H/S/30% CCP)	IS-CCP (H/S/IS-CCP)—In-Situ Loading
**Serie 1**	without additives	without additives	without additives
**Serie 2**	5 kg/t CS 10 kg/T AKD	5 kg/t CS 10 kg/T AKD	5 kg/t CS 10 kg/T AKD
**Serie 3**	5 kg/t CS +10 kg/t AKD 0.025% PPAM	5 kg/t CS + 10 kg/t AKD 0.025% PPAM	5 kg/t CS + 10 kg/tAKD 0.025% PPAM
**Serie 4**	5 kg/t AC+10 kg/t AKD PPA + B = 1:10 cPAM/B = 1:10	5 kg/tAC + 10 kg/t AKD PPA + B = 1:10 cPAM/B = 1:10	5 kg/t AC + 10 kg/t AKD PPA + B = 1:10 cPAM/B = 1:10

Hardwood fibers (H), softwood fibers (S), CCP—calcium carbonate precipitated used for the conventional loading, softwood fibers with 10% in-situ precipitation of calcium carbonate (IS-CCP), cationic starch (CS), alkyl–dimercetene emulsion (AKD), cationic polyacrylamide (PAA) and bentonite (B).
